# The Practice of Hospital Administration

**Published:** 1909-11-27

**Authors:** 


### THE PRACTICE OF HOSPITAL ADMINISTRATION.

### A PRACTICAL BOOK BY A PRACTICAL MAN.*

There is nothing more distressing to a practical man acquainted with the possibilities of hospital administration than the evidence which accumulates with telling force as inspections are made of many hospitals. The purchase of relatively imperfect or useless fittings, the adoption of exploded methods of ventilation, the extravagance almost everywhere manifest with increasing force in hospital buildings, the omissions of practical details and facilities which recent plans too often display, and the multitude of other matters afford melancholy evidence to the expert that something is wrong somewhere. As to plans, the truth is that the Royal Institute of British Architects fails in its duty to the profession and to the public, especially in regard to hospital plans. It has never made it its business to instruct architects efficiently in hospital planning, or, so far as we know, to insist upon candidates for admission to the profession of architecture, having had experience and displaying knowledge of this branch. Its selection of assessors has over and over again proved to be without justification, if we may judge by the results of the awards in several instances. We hold that no man is efficient or capable of discharging the duties of assessor unless he grasps all the conditions under which a competition takes place, and declines the award to every competitor who fails to comply strictly with such conditions. We have on our table as we write evidence that recent assessors can have paid little or no regard to the conditions of the competition, for they have awarded the first place to plans which grievously fail in a hygienic or other sense to fulfil the conditions laid down. There ought to be some method instituted by the Royal Institute of British Architects whereby an assessor who thus fails to do his duty can be brought to book and held responsible, as an accountant would be in the case of an audit. Things cannot go on much longer as they have proceeded lately in regard to competitions for new hospital buildings. Either assessorship must be given up or the Institute will have to assert its authority, so as to prevent an amazing failure on the part of assessors to fulfil conscientiously and adequately the duties imposed upon them. We would counsel the authorities everywhere who have new hospitals to build, if they employ an architect assessor, to associate with him some one who can have no interest whatever in making the award except to the best man, and who is known to possess an intimate knowledge of hospital administration in all departments.

We have been led to these remarks by a perusal of a book by Dr. Mackintosh, superintendent of the Western Infirmary, Glasgow. Dr. Mackintosh is

* "Construction, Equipment and Management of a General Hospital." By Donald J. Mackintosh, M.B., M.V.O., Medical Superintendent of the Western Infirmary, Glasgow. With plans and illustrations. (Edinburgh and Glasgow : William Hodge and Co. Price 10s. 6d. net.)

a practical man, as every page of his book shows, and he represents just the kind of influence and knowledge which is wanted when an award has to be made on several sets of plans for a new hospital. Dr. Mackintosh realises, as we realise, that it is dangerous, and immensely costly, to employ an architect to build a hospital who has no practical knowledge of its interior working and administration. Architects fail, as a rule, as Dr. Mackintosh points out, to grasp that the theory of hospital construction is a living thing. That is to say, it is never possible at any stage to say that a climax of excellence and completeness has been reached. The failure to appreciate this fact has produced a number of striking failures, as represented by recent hospital buildings of importance. What are we to say of a man who spends hundreds of thousands of pounds and shows so little apprehension of the requirements of a great hospital that he places his operation theatres in a position which compels every patient operated upon to be brought out into the open air, and possibly to traverse some distance in it for some minutes? How many deaths may result from this one failure to appreciate realities, without possibly the architect ever realising the innumerable deaths which may be justly laid in large measure at his door. When the staff and the administration realise a fact like this, how can they, after spending, say, half a million of money, be called upon to provide thousands of pounds more to put things right? We propose to pursue this subject further later on, and will here content ourselves with the statement that we hope that architects who sit down to plan portions of a hospital will from this date at least make themselves fully acquainted with the practical details which Dr. Mackintosh gives with a completeness that is worthy of all praise.

A book like this should be tested not by its literary but by its practical merits. It is essentially a book of practice, and in that sense it is probably the most useful book of the kind which has yet been published. The readers of The Hospital will be familiar with much which the book contains. Beginning at the hospital entrance, Dr. Mackintosh describes the gate-house, or admission block, which first took practical shape at the Royal Infirmary, Glasgow. A study of that block and its erection marks a new departure in hospital construction. It should afford food for thought to every administrator in charge of a great hospital or kindred institution. Passing on, Dr. Mackintosh describes separately the medical, surgical, and special ward units. He gives a number of photographs, and makes many good points. Of course, in regard to the planning and many of the fittings, readers must remember that Dr. Mackintosh has been brought up under, and represents, the Scottish system of administration. This materially differs from that in other countries, from the circumstance that each unit has to be complete in the sense that accommodation must be provided in connection with it for the resident staff who are responsible for the work there. This system will be better understood after a study of the plans and a perusal of chapters v. and vi. dealing with the medical officers, the nursing and the wardmaids' staff for each unit. Terse, instructive, and understandable may be applied to the description of each department in this book. These descriptions are complete, too—a most important fact in the present day, where there is too much tendency for one man to copy another man's work, forgetting that this plan often affords the most convincing evidence of the unfitness of the last author of such a book. Instead of giving a lot of plans of a number of hospitals, Dr. Mackintosh gives units which are complete and up-to-date. That is the only system, after all, which enables a practical man with knowledge to teach others how to acquire it.

We would specially commend the out-patient and laundry departments or units, with the plans and particulars given, to all who are interested in the administration of hospitals. There are points in this out-patient department unit which are not to be met with in any other out-patient department in the world. They represent an amount of thought, knowledge and ability rarely met with, and still more rarely put into practice. We have not space to indicate these points in detail, but they must be apparent to the trained administrator who takes an intelligent interest in his or her business. To members of the medical profession this book should commend itself, for the reason that it will give every member of an honorary staff an opportunity of studying practical details which cannot fail to make him more capable of criticising an architect's plans and expressing an opinion of some value upon them. At present the honorary staff seldom exhibit a practical knowledge of hospital construction, and its absence is often replaced by an individual desire to enforce the adoption of fads, many of which have been exploded in the experience and working of other hospitals. This circumstance has contributed as much as anything to many disappointments which have resulted from the erection of new hospitals; and the absence of one controlling mind to take charge of the whole of the works has had equally disastrous results. We are glad, too, that Dr. Mackintosh has added a chapter on the provision for nervous and incipient mental diseases. What he says contains much practical good sense, and though it has been said before in our columns and elsewhere, it is well that it should find a permanent place in a practical book like that under review.

Attached to the book are a number of appendices, including an outfit for the operation theatre and inventories for a ward of eighteen beds of linen, furniture, crockery, and cutlery, with the probable cost. We have examined these inventories with some interest, and wonder why an inventory for children's wards, for nurses, and for servants is not given. We also notice that the sizes of the sheets and pillow-cases are omitted. Again, whereas the allowance for linen seems generous and adequate to the demands of asepsis, the prices given seem to us to be remarkably low, if the best quality of goods only is purchased, as it should be purchased for use in a voluntary hospital. There is nothing so cheap as the best goods for use in a hospital, a fact which is too often forgotten or disregarded. The list of furniture for the wards is restricted, and we do not gather—though in the absence of the size we can only judge by the cost—

that a table of adequate proportions is included. We would further suggest that it is a very attractive and useful feature to have a portable couch or trolley in each ward. There is a good pattern of such a trolley in use at the Huddersfield Infirmary. No poison cupboards are included in the ward lists, which we presume means that they are kept outside the wards.

We would suggest that in future editions of this book the plans should be printed in the book itself, and not at the end on thin paper, which is sure to get torn, and which for practical purposes is very difficult to handle. The same remark applies to Appendix No. VII., which deals with the nursing staff. The information given as to the conditions under which medical and surgical appointments are held in the principal hospitals of the United Kingdom will be welcomed. It shows, in view of modern developments, on the surgical side especially, that in many hospitals a revision of these conditions would seem to be called for. Taken altogether this is a practical and useful book, which should find its way on to the table of everyone who is engaged practically in the administration of an important British hospital. We have little doubt that the book will attain an even wider circulation, for much of the practical information it contains has a general application, and should prove useful to hospital managers all the world over.

